# The impact of the Himalayan aerosol factory: results from high resolution numerical modelling of pure biogenic nucleation over the Himalayan valleys†

**DOI:** 10.1039/d4fd00171k

**Published:** 2025-03-19

**Authors:** Giancarlo Ciarelli, Arineh Cholakian, Manuel Bettineschi, Bruno Vitali, Bertrand Bessagnet, Victoria A. Sinclair, Johannes Mikkola, Imad el Haddad, Dino Zardi, Angela Marinoni, Alessandro Bigi, Paolo Tuccella, Jaana Bäck, Hamish Gordon, Tuomo Nieminen, Markku Kulmala, Douglas Worsnop, Federico Bianchi

**Affiliations:** a Institute for Atmospheric and Earth System Research/Physics, Faculty of Science, University of Helsinki 00014 Helsinki Finland giancarlo.ciarelli@helsinki.fi; b LMD UMR CNRS 8539, ENS, École Polytechnique, Institut Pierre Simon Laplace (IPSL), Route de Saclay 91128 Palaiseau France; c Sustainable Development and Energy Sources Department, Ricerca sul Sistema Energetics – RSE S.p.A. Via R. Rubattino, 54 20134 Milan Italy; d International Center for Integrated Mountain Development (ICIMOD) Lalitpur Nepal; e Laboratory of Atmospheric Chemistry, Paul Scherrer Institute 5232 Villigen Switzerland; f Department of Civil, Environmental and Mechanical Engineering, University of Trento Trento Italy; g Center Agriculture Food Environment – C3A, University of Trento Trento Italy; h Institute of Atmospheric Sciences and Climate, National Research Council of Italy (ISAC-CNR) 40129 Bologna Italy; i Dipartimento di Ingegneria “Enzo Ferrari”, Università di Modena e Reggio Emilia 41125 Modena Italy; j Department of Physical and Chemical Sciences, University of L’Aquila L’Aquila Italy; k Center of Excellence in Telesensing of Environment and Model Prediction of Severe Events (CETEMPS), University of L’Aquila 67100 L’Aquila Italy; l Institute for Atmospheric and Earth System Research/Forest Sciences, Faculty of Agriculture and Forestry, University of Helsinki FI-00014 Helsinki Finland; m Department of Chemical Engineering, Carnegie Mellon University Pittsburgh PA USA; n Center for Atmospheric Particle Studies, Carnegie Mellon University Pittsburgh PA USA; o Department of Physics, Faculty of Science, University of Helsinki Helsinki Finland

## Abstract

Observational data collected in December 2014 at the base camp of Mount Everest, Nepal, indicated frequent new particle formation events of pure biogenic origin. Those events were speculated to be controlled by the along-valley winds forming in the valley connecting the Indo-Gangetic plain to the observational site, the Nepal Climate Observatory-Pyramid. The valley winds funnel highly oxygenated organic molecules of biogenic origin to higher elevations where they nucleate. The mechanism was referred to as “The Himalayan aerosol factory”. Its geographical extent and climate implications are currently unknown. In view of this, we conducted numerical chemical model simulations to corroborate the presence of the mechanism, and to quantify its geographical extent. Our numerical simulations confirmed that biogenic emissions located in the valleys can be converted into ultra-low volatility organic compounds, transported to the observational site by the along-valley winds, and therein nucleate. The overall time scale of the process, from the release of biogenic emissions to the conversion to ultra-low volatile organic compounds to the arrival time at the observational site, was found to be around 4 hours, consistent with the predicted along-valley winds intensity and the geographical distribution of biogenic emissions. A first estimation of the maximum injection height of biogenic particles, and highly oxygenated organic molecules, indicated the presence of efficient nucleating gases and biogenic particles at an elevation as high as 5000–6000 m a.s.l. These results suggest that the Himalayan chain, under specific weather conditions, is a main contributor to the biogenic aerosol loads in the free troposphere. Considering these findings, field campaigns, especially at the entrance of the valley’s floors, and research consortia supporting atmospheric research in Asian mountain regions, are highly encouraged.

## Abstract 2

A schematic of the “Himalayan aerosol factory”. Courtesy of Sole Lätti (https://kuvittajat.fi/).

## Graphical abstract

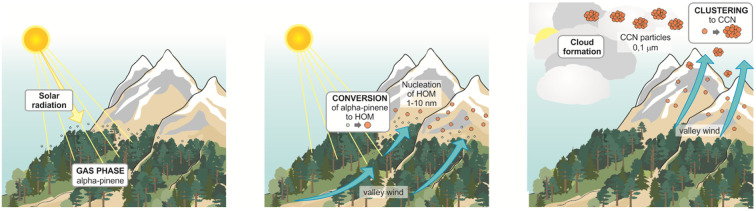

## Introduction

Trees emit biogenic volatile organic compounds (BVOCs) that are injected in the shallower layers of the Earth’s atmosphere.^1^ Once in the atmosphere, BVOCs are oxidized by atmospheric oxidants like the hydroxyl radical (OH), ozone (O_3_), and nitrate (NO_3_) leading to the formation of new molecules with higher or lower volatility compared to their parent precursor.^2^ While high volatility organic gases will reside in the gas phase and be removed by scavenging processes, the low volatility organic gases can contribute to the formation of new particles *via* nucleation, and to the enhancements of the aerosol mass *via* condensation. Both processes, nucleation and condensation, are referred to in the literature as secondary formation mechanisms and apportioned as such in most numerical models.^3^ A unified description seeking to depict the chemical and physical transformations of those molecules is a great challenge. The massive amount of different emitted organic gases, both from biogenic and anthropogenic sources, complicates the exact characterization of each of the single chemical pathways they will undergo when in the atmosphere. In oxidation experiments of α-pinene (C_10_H_16_), a BVOC emitted from the *Pinus sylvestris* (Scots Pine), mass spectrometry analysis of the ionized organic products can reveal a broad range of molecules, from small ion fragments at around and above 100 mass to charge ratio (*m*/*z*), to small, oxidized products at around 120–150 *m*/*z* to more complex molecules at 300 *m*/*z* and far below.^2^ Small, oxidized products such as aldehydes, ketones are associated with ozonolysis first oxidation products, following the double bond attack of α-pinene by O_3_. Historically, these products have been used to fit the mass yields of biogenic secondary aerosols (BSOAs) in chemical transport models, categorizing them in an effective saturation vapour pressure (C*) range between 0.3 and 300 μg m^−3^. Molecules in this range are referred to as Semi Volatile Organic Compounds (SVOCs), whereas others in a higher volatility range are referred to as, for example, Intermediate Volatility Organic Compounds (IVOCs).^4^ They cover the wide range of organic aerosol concentrations usually encountered at ambient conditions and they can exist in both the gas and the condensed phases at equilibrium. Large complex molecules at 300 *m*/*z* and below, however, are produced *via* different chemical pathways, *i.e.* autooxidations, which involves the peroxyl radical (RO_2_) H-shift and O_2_ addition, a process which is strongly dependent on temperature and nitrogen oxide (NO) levels.^4^ Those molecules can have a C* as low as 3 × 10^−8^ μg m^−3^; they can spark new particles’ formation (NPF) and sustain their subsequent growth to larger diameter sizes. It has been shown that molecules with C* as small as 3 × 10^−8^ μg m^−3^, referred to as Ultra Low Volatility Organic Compounds (ULVOCs), are efficient initiators of NPF, whereas others with higher volatility are fundamental to sustaining their growth to larger diameters.^5^ In the sub-3 nm particles range the growth is sustained by so called Extremely Low Volatility Organic Compounds (ELVOCs) having a C* < 3 × 10^−5^ μg m^−3^ since SVOCs and Low Volatility Organic Compounds (LVOCs) are likely not able to overcome the well-known Kelvin effect which counteracts the mass flux transfer of those molecules toward the nanoparticle.^6^

The Himalayas region hosts approximately 19 000 plant species and is characterized by monsoon-driven vegetation in the eastern regions and drier and cooler climate vegetation in the western part.^7^ In December 2014, an intensive measurement campaign was conducted at the Nepal Climate Observatory-Pyramid (NCO-P, 5079 m a.s.l.), located at the base camp of Mount Everest in Nepal. The instrumentation, which comprised of Atmospheric Pressure Interface Time-of-Flight Mass Spectrometers (APi-TOF, Tofwerk AG), a Particle Size Magnifier (PSM, Airmodus) and a Neutral Cluster Air Ion Spectrometer (NAIS, Airel), allowed identification of the NPF events with exact information on the particles’ size distribution and on their chemical footprint. The results revealed an unprecedented new mechanism consisting of intensive NPF events of pure biogenic origin which was initiated by the transport of biogenic precursors by along-valley winds. Dispersion model calculations based on the Weather and Research Forecast (WRF) model driven with synthetic tracers, *i.e.* WRF-FLEXPART, indicated that those particles can travel hundreds of kilometres beyond the observational site under specific synoptic conditions.^8^

In this study, we seek to provide explicit chemical numerical evidence of such a mechanism by conducting state-of-the-art chemical transport model simulations at a spatial resolution suitable for mountain ecosystems, *i.e.* down to 1 km. The study was also designed to explore the extent to which such a mechanism affects the aerosol load in the free troposphere above the Himalayas.

## Methods

The WRF-CHIMERE (v2020r3)^9^ model was set-up with a 4-domain nested configuration following similar set-ups as in our previous applications,^8,10,11^*i.e.* from 27, to 9 to 3 to 1 km horizontal resolution, and with the inner domains slightly reduced in their horizontal geographical extent to minimize the computation cost of the numerical simulations. Since the WRF settings are almost identical to our previous applications, we do not report them here for the sake of brevity. The fourth inner-most domain at 1 km resolution was operated on an adaptive time step, which was key to satisfying the Courant–Friedrichs–Lewy (CFL) condition and to avoid excessive vertical velocity in the vicinity of Mount Everest. The gas-phase chemistry was driven with the SAPRC-07 chemical mechanism^12^ coupled with the volatility basis set^13,14^ for the thermodynamic treatment of the organic material, and with several updates for the application of interest as the reader will find in the next section. Aerosol particles are distributed into twenty bins from 1 nm to 10 μm in diameter on a logarithmic sectional distribution, and emissions of BVOCs calculated with the Model of Emissions of Gases and Aerosols from Nature (MEGAN) v2.1 using the vanilla vegetation data of the model.^15^ Boundary conditions of O_3_ and dust emissions were taken from the climatological simulations of LMDz-INCA3 (ref. 16) and the Goddard Chemistry Aerosol Radiation and Transport (GOCART) model,^17^ respectively. For O_3_, concentrations were corrected using data from NCO-P to minimise the uncertainties in the oxidant’s levels. In light of the observational evidence, no anthropogenic influence was accounted for in the numerical simulations presented here, apart for a fixed background value of sulphur dioxide (SO_2_), *i.e.* around 0.1–0.5 ppb, as indicated by the observational data, and a preexisting minimal amount of organic material reaching 0.1 μg m^−3^.

**Fig. 1:**
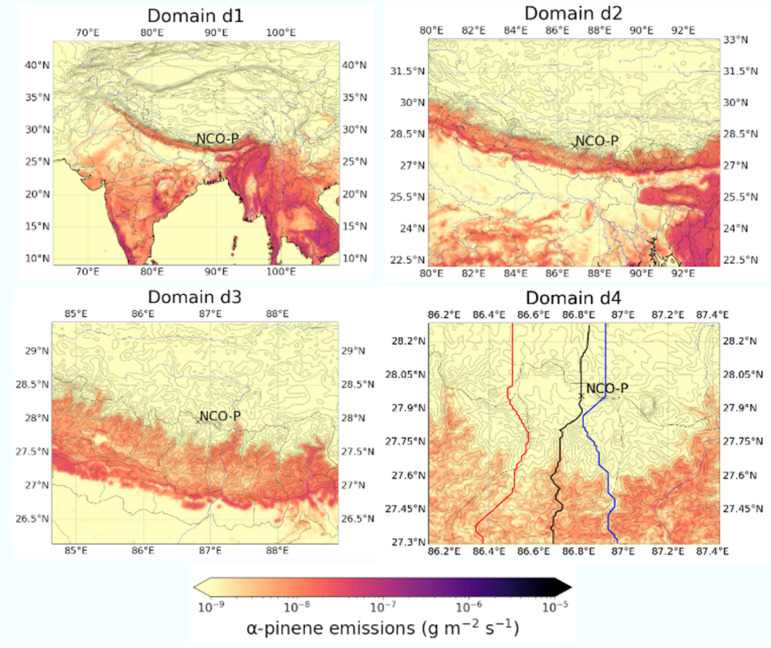
Adopted model’s domains. d1 to d4 indicates domains at 27, 9, 3, and 1 km resolution, respectively. The red and blue lines are the western and eastern valley ridges along the Khumbu valley, respectively. The black line follows the Khumbu valley floor profile all the way from the NCO-P observational site to the Tibetan Plateau.

Simulations were performed from the 16th to the 21st of December of 2014 with the first day of the simulation neglected as spin-up. Fig. 1 illustrates the extent of the 4 domains with overlapped average biogenic emissions of α-pinene as estimated by MEGANv2.1. Notice the massive streak on d1, extending from about 95E to 70E, home of the Himalayan valleys.

## Model development of nucleation and condensation schemes

The work is a result of a collaboration between the InterAct group at the University of Helsinki, the Institute for Atmospheric and Earth System Research (INAR) and the members of the developer team of the WRF-CHIMERE model at the Laboratoire de Météorologie Dynamique (LMD) of the Institut Pierre Simon Laplace (IPSL), Paris. Model development and evaluation was first initiated with a comprehensive study using the WRF-CHIMERE (v2020r2) model over the Finnish Boreal Forest, collecting observation data from the SMEAR-II station located in Hyytiälä.^18^ The site allows for a comprehensive evaluation of the model’s performance, from meteorological parameters, to emissions, to chemical conversion and removal mechanism. A second application was run on complex terrain, *i.e.* the Northern Italian Apennines, with a focus over the Mount Cimone global atmospheric watch (GAW) station to probe the model’s behaviour over mountain regions.^10^ Both studies were directed at ensuring the quality closure of the total organic and biogenic organic aerosol masses in areas under substantial influence of BVOCs but different orography, with positive outcomes in both applications. The studies confirmed the importance of aging reactions of biogenic secondary organic vapours, a parameter historically probed in standard CTMs (chemical transport models) applications,^19,20^ as well deficiencies in isoprene (C_5_H_8_) emissions over the European boreal forest when the vanilla vegetation data of MEGAN was used, *i.e.* emissions were largely overpredicted with important consequences on the gas-phase chemistry, such as suppressing secondary biogenic secondary aerosols formation by scavenging away OH radicals from α-pinene oxidation.

We extended here the approach to treat the organics in the WRF-CHIMERE model by (i) including the formation, *i.e.* yields, of ULVOCs and ELVOCs from α-pinene oxidation, (ii) including the pathways of neutral, ion-induced pure biogenic nucleation, and sulfuric acid–organic nucleation, and (iii) by including a kelvin barrier during the gas to particle mass transfer rates calculations. Additionally, (iv) we updated the neutral and ion-induced sulfuric acid–water nucleation pathway based on more recent literature data.^21^

We first initiate the model development with the inclusion of two additional surrogate gas-phase species, ULVOCs and ELVOCs.

For ULVOCs:
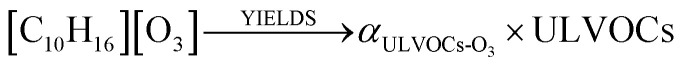
with:
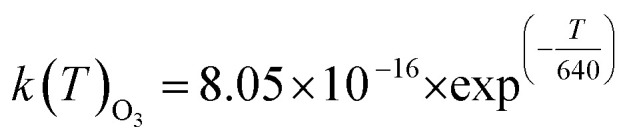
where *k*(*T*)_O_3__ is the temperature-dependent rate constant equation for O_3_ in the Arrhenius form, and *α*_ULVOCs-O_3__, the mass yield of ULVOCs. In Dada *et al.*^5^ molar yields of ULVOCs were estimated to reach 0.25%.

The yields were applied to the O_3_ oxidation pathway since only the total yields were reported in Dada *et al.*^5^ The final concentration of ULVOCs (molecules^−1^ cm^−3^) is assumed to be at the steady state:
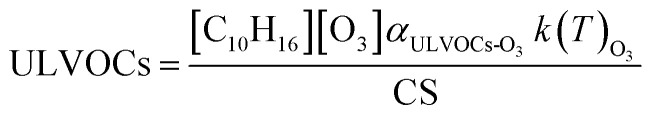
where CS is the condensational sink calculated as:
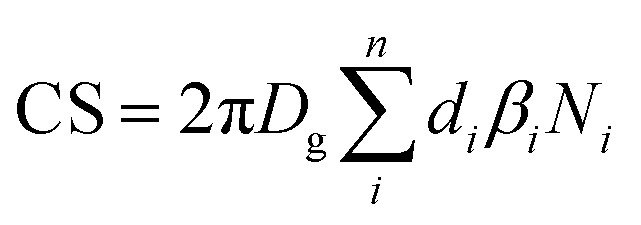
with *D*_g_ being the diffusion coefficient, assumed to be 1 × 10 ^−5^ m^2^ s^−1^, of the organic gas in the air, *d* is the particle diameter, *β*_*i*_ the Fuchs–Sutugin correction coefficient and *N*_*i*_ the particle number concentration in the *i*th bin, with *n* = 20.

For ELVOCs:
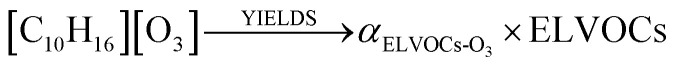

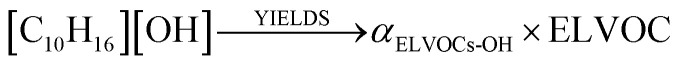
with:
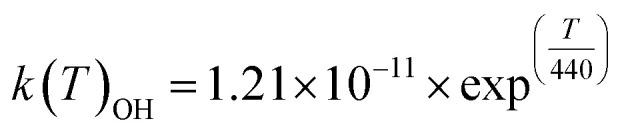
where *k*(*T*)_OH_ is the temperature-dependent rate constant equation for OH in the Arrhenius form, and *α*_ELVOCs-O_3__ and *α*_ELVOCs-OH_, the mass yields of ELVOCs for O_3_ and OH, respectively. In Jokinen *et al.*^22^ molar yields from ozonolysis of α-pinene experiments were estimated to be 3.4 and 0.44%, for O_3_ and OH, respectively. These compounds were assigned an effective saturation vapour pressure of 1 × 10^−5^ μg m^−3^ in the volatility basis set space. Organic material in the 1 × 10^−2^–1 × 10^6^ μg m^−3^ effective saturation vapour pressure range was already parametrized in the vanilla version of the model.^14,23^

ULVOCs were used to drive the pure biogenic nucleation mechanism, both neutral and ion induced. Following Kirkby *et al.*:^24^
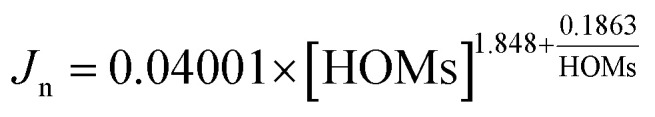

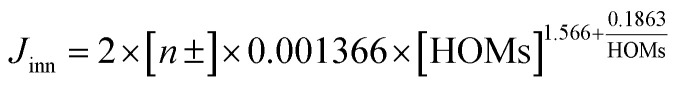
where *J*_n_ and *J*_inn_ are the neutral and ion-induced nucleation rate and HOMs the total amount of highly oxygenated molecules. In our study the HOMs in the above equation equals the amount of ULVOCs, being the only ones responsible for the initialization of the new particle formation. Therefore the overall equation was rescaled by a factor of 3.6 as proposed in Zhao *et al.*^25,26^

As in Kirkby *et al.*,^24^ the ion concentration is calculated as:
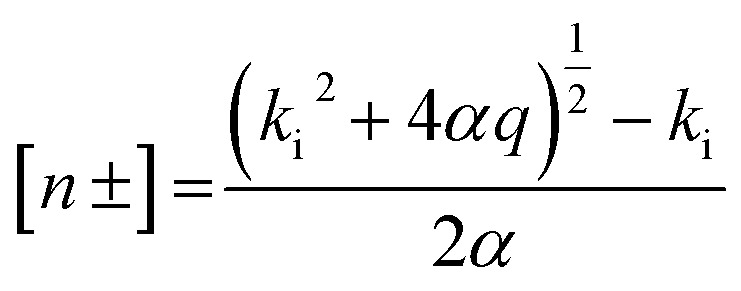
where *q*, (in cm^−3^ s^−1^) is the ion-production rate set in the model to 1 cm^−3^ s^−1^, *k*_i_ the ion loss rate including losses by condensational sink and ion-induced nucleation and *α* the ion–ion recombination rate parametrized as in Brasseur and Chatel.^27^ The ion losses rates by condensational sink on pre-existing particles have been parametrized using a linear ion loss rate roughly following Hoppel and Frick.^28^ A temperature dependence function is applied on the nucleation rate defined above as in Zhao *et al.*:^25^
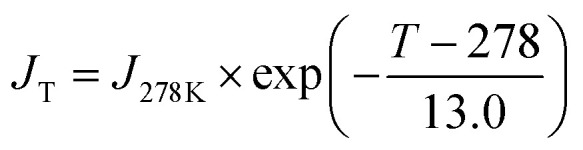


Two additional nucleation pathways have been included in the model, *i.e.* the sulfuric acid–organic nucleation pathway as in Riccobono *et al.*,^29^ and the neutral and ion-induced sulfuric acid–water nucleation pathway as reported in Dunne *et al*.^21^ Since those are not the central focus of this study, and their contributions were found to be negligible in this specific application, as discussed later, we do not report their explicit formulation here.

The condensing mass fluxes are based on the kinetic of condensation approach already available in WRF-CHIMERE, which is mostly based on the work of Wexler and Seinfeld^30^ and updated for this application to prognostically account for the Kelvin effect, since WRF-CHIMERE did not account for it.

For each single *j*th gas-phase species, the model calculates the particle mass gain coefficient in the *i*th bins as:gain(*i*, *j*) = 4π*R*_p,*i*_*D*_g_*N*_*i*_*f*(Kn_*i*_,α)where *R*_p,*i*_ is the radius of the particles in the *i*th size bin, *D*_g_, the gas diffusion of the organic gas coefficient in air, *N*_*i*_ the total number of particles in the *i*th bin, *f*(Kn_*i*_, *α*) the Fuchs and Sutugin transition regime correction factor^3^ which is a function of the Knudsen number (Kn_*i*_), and *α* the mass accommodation coefficient. The dependencies in the above equation on the specific *j*th gas-phase component arise in our formulation from the Knudsen number embedded in the *f*(Kn_*i*_, *α*) function, since both the *D*_g_ and *α* are set to the unique values of 1 × 10^−5^ m^2^ s^−1^ and 0.5, respectively.

The mass transfer driving force from gas to particles is ignited when:
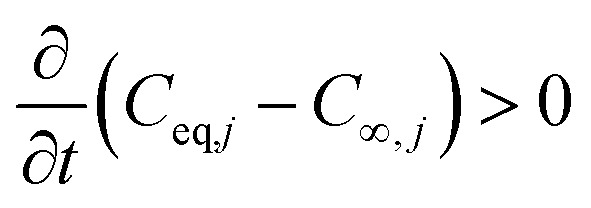
where *C*_∞,*j*_ is the bulk gas-phase concentration of the *j*th species (sometimes referred to as the concentration “far away” from the particle), and *C*_eq,*j*_ is the is the equilibrium gas-phase concentration of the *j*th species with the particle-phase, *i.e.* at the interface of the particle.

The differential equation of the mass transfer rates for particles becomes:
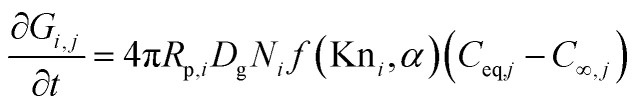
with *G*_*i*,*j*_ in molecules per cm^3^ per s (we omit to report the differential equation mass transfer rates for gases, which are mainly reverse in sign). The numerical solution of the above equation is the primary bottleneck in terms of computational time for CTMs. The reader might have noticed that the above formulation lacks the explicit dependence of the *i* index on the of the *C*_eq,*j*_ term, *i.e.* the Kelvin effect. Therefore, a Kelvin barrier was imposed in the model at 3 nm for SVOCs and LVOCs compounds, inhibiting the condensation of such compounds on sub-3 nm particles, and allowing only ELVOCs to growth freshly nucleated particles, which are injected in our model at the 2 nm diameter size bin, the closest bin to the 1.7 nm diameter in the logarithmic sectional distribution.

In a way, after a nanoparticle is born, it seems that it would rather prefer to be left alone as it tries to make its surface area-to-volume ratio as large as possible, shielding itself against the most abundant primary and secondary organic gases present in the Earth’s atmosphere, the S-I-VOCs. Only the least abundant organic gases, *i.e.* the ELVOCs, are somehow capable of seducing it, and their condensational journey together can start. It is a trap: after eventually reaching cloud condensation nuclei size in supersaturated air, it will be removed from the atmosphere (and in a much shorter time than if it was left alone, or if it was lucky enough to be born in the peacefulness of the free troposphere). After all, “all those particles will be lost in time, like tears in rain.” (*cf.*^31^).

Following absorptive partitioning theory proposed by Pankow *et al.*,^32^ and largely the formalism therein, *C*_eq,*j*_ is:
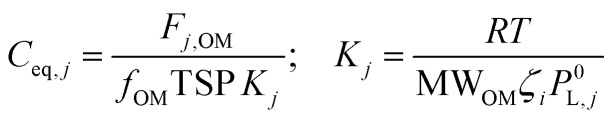
where *F*_*j*,OM_ is the particle phase concentration of the *j*th compounds in the organic matter phase in μg m^−3^, TSP is the concentration of the total suspended particles in μg m^−3^, and *f*_OM_ is the weight fraction of the absorbing organic matter in TSP. *K*_*j*_ is the Pankow coefficient in units of m^3^ μg^−1^ and represents the gas to particle partitioning constant, *P*^0^_L,*i*_ is the saturation vapour pressure of the pure *j*th compound in atm at 300 K, MW_OM_ is the mean molecular weight of the absorbing organic material in g mol^−1^ and *ζ*_*i*_ the activity coefficient of the *j*th compound in the organic matter phase, assumed to be 1 for all the *j*th compounds. *R* and *T* are the gas constants and temperature of interest in Kelvin, respectively, with *R* expressed in J mol^−1^ K^−1^. We would like to emphasise that in our discussion, the term saturation vapour pressure (*P*^0^_L,*i*_) and equilibrium vapour pressure (*C*_eq,*j*_) are not interchangeable, as the equations above nicely show. That equation requires an iterative solution. This is because the total amount of TSP in the organic matter (denominator in equation above) controls the partitioning, but the amount of the specific *j*th compound in that phase is initially unknown (the particle phase *F*_*j*,OM_ in the numerator above). This *analogia entis* can also be resolved for each *j*th compound once an appreciable amount of organic material is present, *i.e.* by relaxing the need for an iterative solution:*f*_OM_TSP → ∞ ⇒ *K*_*j*_ → constant

Finally, the standard Clausius Clapeyron equation adjusts the effective saturation vapour pressure based on local temperature assuming a vaporisation enthalpy of 30 kJ mol^−1^ for all the *j*th compounds.

## Results

**Fig. 2:**
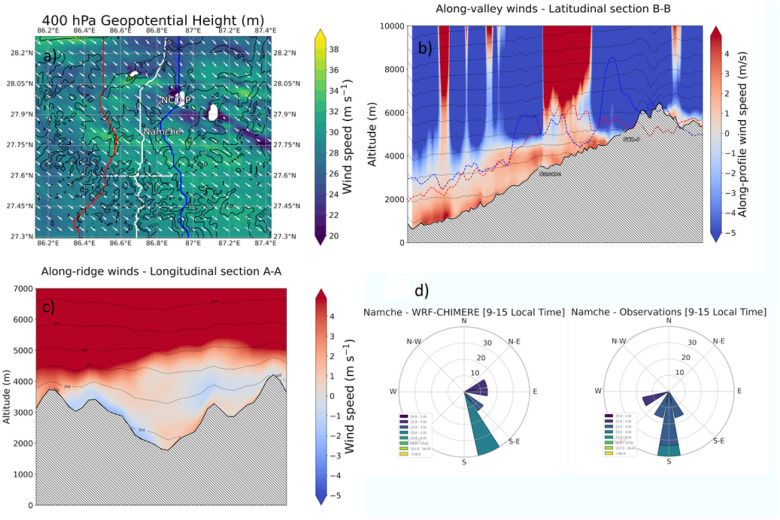
(a) Modelled 400 hPa geopotential height (m), (b) along-valley winds, (c) cross-valley winds (m s^−1^) with overlapped potential temperature (K). (d) Wind roses of model and observations data obtained from the Namche stations. All data as 9–15 local time averages. Results are shown for the inner domain at 1 km resolution.

Fig. 2 reports the modelled 400 hPa geopotential height, the along-valley and cross-valley wind profiles and the comparison with observational data obtained from the Namche station located at approximately midway between the valley entrance and the Tibetan plateau.

The presence of the typical valley circulation during daytime hours is clear, with along-valley and cross valley winds forming in the valley at around 2 to 4 m s^−1^.

The analysis also indicates the presence of a strong synoptic circulation superimposed on the valley, and with a predominant northern-westerly direction. Comparisons with observational data show that the model captures those patterns, with minor inevitable discrepancies in terms of wind intensity and directions (Fig. 2). These results are in line with our previous studies^8,11,18^ and the reader is recommended to refer to those studies for a comprehensive discussion on the meteorological model evaluation over complex terrain.

**Fig. 3:**
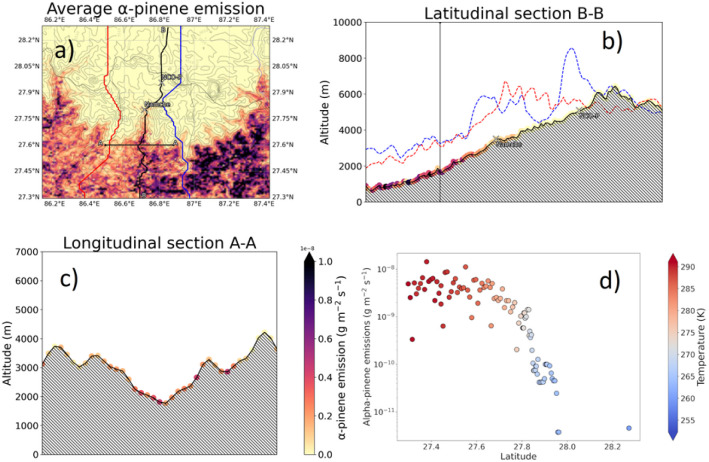
(a) Average α-pinene emissions over the inner resolution domain (1 km) and (b) along the latitudinal (B–B) and (c) longitudinal (A–A) sections of the Khumbu valley. Dots along the profiles indicate the location and intensity of the emissions (g m^−2^ s^−1^). The dependence of the α-pinene emission on the latitudinal section is additionally reported as scatter plots, and colour coded by the temperature (K) in the lower right panel, (d). Data have been averaged between the 17th and 21st of December 2014.

Fig. 3 illustrates the modelled α-pinene biogenic emissions as generated with MEGANv2.1. The figure aesthetic matches the one already presented in Fig. 2 to facilitate the comparison of the different fields as the discussion proceeds. Emissions exhibit a vigorous south to north gradient, with roughly a 3 orders of magnitude difference between the valley entrance and the NCO-P. This pattern is driven by both the along-valley temperature gradient (Fig. 3), as well as the along-valley vegetation gradient (not shown). Such a pattern is not only observed along the latitudinal section following the valley floor, *i.e.*, latitudinal B–B section, but also along the longitudinal section A–A, which was placed at the tipping point of the emission gradient. This is at a latitude of around 27.6N, where biogenic emissions decline by a factor of 10^3^ over a distance of ∼20 km and over an elevation gain of ∼2000 m. At NCO-P, emissions have further declined by another half order of magnitude compared to Namche (“isolated” points at the bottom of the α-pinene-latitude plot in Fig. 3). Based on those results, we concluded that the large fraction of α-pinene emissions in the Khumbu valley are located at least 40 km south of the NCO-P, with the northern regions being free from biogenic, organic, influences because of the presence of bare terrain, and deserts (where no BVOCs are present).

We proceed now with the discussion of the modelled highly oxygenated organic molecules along the Khumbu valley and their role on new particle formation at NCO-P. For the analysis, we selected two days out of the six we have computed (one being discarded as spin-up), *i.e.* the 17th and 18th of December. This is for two reasons: (1) during those days, the patterns of the along-valleys winds were best reproduced in the models. Even though the daytime components of the along-valley winds are always captured in all days of our simulations, the transition between up-valley and down-valley winds in the model is the smoothest and at is best during those two days, *i.e.* less noisy,^8^ (2) these two days allow to a discernment between a non-NPF event (17th of December) and NPF event (18th of December) with the 18th being one of the days when NPF intensity is at its maximum during the whole campaign.^8^

**Fig. 4:**
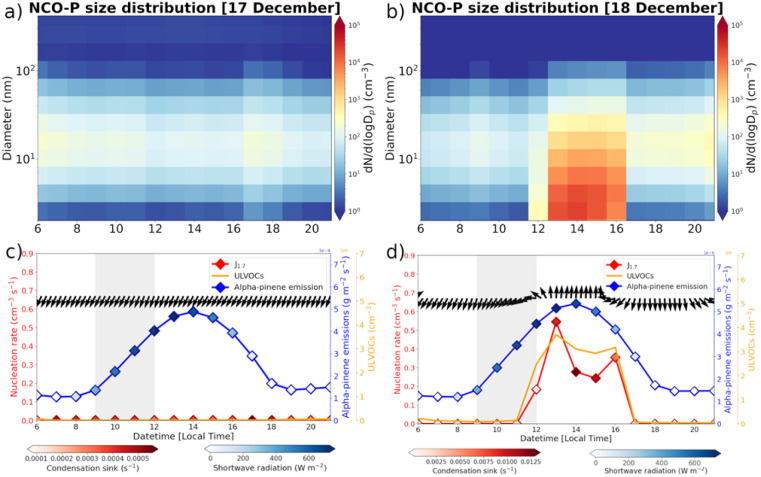
Upper-panel: modelled particle number size distribution. 17th of December on the left (a), and 18th of December on the right (b). Lower-panel: α-pinene biogenic emissions at the A–A section (blue line) colour coded by short-wave radiation (W m^−2^), ULVOCs concentrations (molecules per cm^3^), *J*_1.7_ nucleation rate (particles per cm^3^ per s) colour coded by condensational sink (molecules per s), and wind direction in black arrow. 17th of December on the left (c), and 18th of December on the right (b).

The lower panel of Fig. 4 reports the intensity of biogenic emissions at the A–A section, 40 km south of NCO-P, together with the shortwave radiation and modelled wind directions. Additionally, the concentrations of ULVOCs and the *J*_1.7_ modelled nucleation rate are reported and colour coded with the values of the modelled condensational sink (CS, in molecules per s) at NCO-P. Emissions in the valleys start to surge at 9 local time and peak at around 14 local time. On the 17th of December northerly winds are predominant at NCO-P, as indicated by the black arrows in the lower-left panel in Fig. 4, with ULVOCs at NCO-P nowhere to been seen. The nucleation rate is zero, and no NPF event was predicted by the model, as in the measurements. A constant band of a few hundreds of particles could be seen, caused by the general background recirculation inside the domain.

**Fig. 5:**
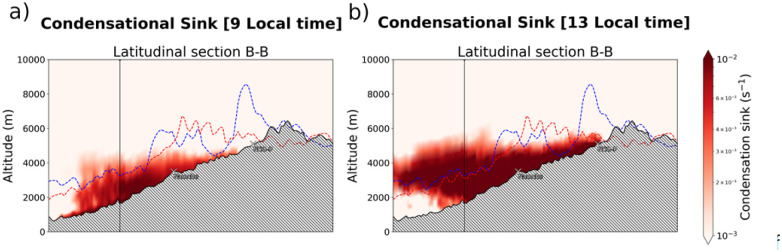
Condensational sink (molecules per s) at (a) 9 and (b) 13 local time of the 18th of December 2014 along the latitudinal section of the Khumbu valley.

An identical analysis is reported on the right side of Fig. 4, but for the 18th of December. On this day, the typical diurnal pattern of the along-valley mountain circulation can be observed in the black arrows, with progressive alternation from northerly to southerly directions as the thermally driven winds in the valley continue to develop until 15 local time, when it starts to reverse following the decline in radiation. Now, ULVOCs molecules at concentrations around 2 − 3 × 10^6^ molecules per cm^3^ can be observed as the along-valley winds are steadily on course along their southerly direction, triggering a NPF event at 0.5 particles per cm^3^ per s. At the start of the nucleation event, the modelled CS were below 0.002 molecules per s and this rapidly increases as nucleation and condensation growth proceed (dark red colours). The development of the CS is additionally reported in Fig. 5, showing the CS along the B–B section, before and at the starting time of the NPF event at NCO-P.

A NPF event is clearly observed in the modelled size distribution, in contrast to the 17th of December. Consistent with observational evidence, the modelled starting time of the NPF event does not correlate with the shortwave radiation, but rather with its precursor’s arrival time at NCO-P, *i.e.* the ULVOCs. Overall, there is about 4 hours delay between the surging of α-pinene emissions at the A–A section, in the middle of the valleys, and the arrival time of the ULVOCs at NCO-P, indicated in the model with the grey shaded area (Fig. 4). At about 3 m s^−1^ (Fig. 2), in 4 hours the air masses would have covered roughly 40 km, which is indicatively the location of the high intensity biogenic emissions, *i.e.* section A–A in Fig. 3.

**Fig. 6:**
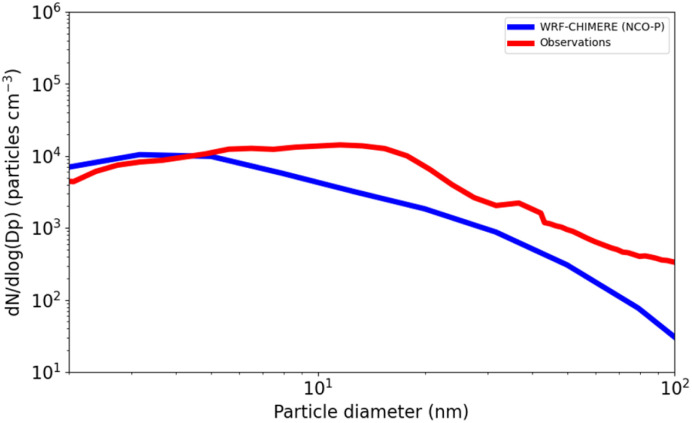
Observed (red lines) and modelled (blue lines) average size distribution (6–21 local time) at NCO-P. Measurement data from NAIS (2–40 nm) and SMPS (above 40 nm).

Fig. 6 shows the modelled and observed particle number size distribution over the 6–21 local time at NCO-P. The measurements were reconstructed by combining NAIS measurements between 2 to 40 nanometres and SMSP data from 40 nm on, as in Bianchi *et al.*^8^ The modelled size distribution aligns well with the measured one, with increasing biases as particles get larger than 5 nanometres, and with larger differences at around 100 nanometres.

The reasons might be numerous, with underestimated particle growth being the most likely culprit. However, given the complexity of the location, the model not only has to properly sustain the particles growth with appropriate emissions and chemical conversion to ELVOCs, *i.e.* the secondary formation, but, and perhaps more vital for this application, also the funnelling of highly oxygenated molecules all along the intricate turns of the Khumbu valley for about 40 km all the way to NCO-P. This is a challenge in CTMs, especially if compared to large convective motions where CTM applications have been proved insensitive to an increase of horizontal resolution.^25^

**Fig. 7:**
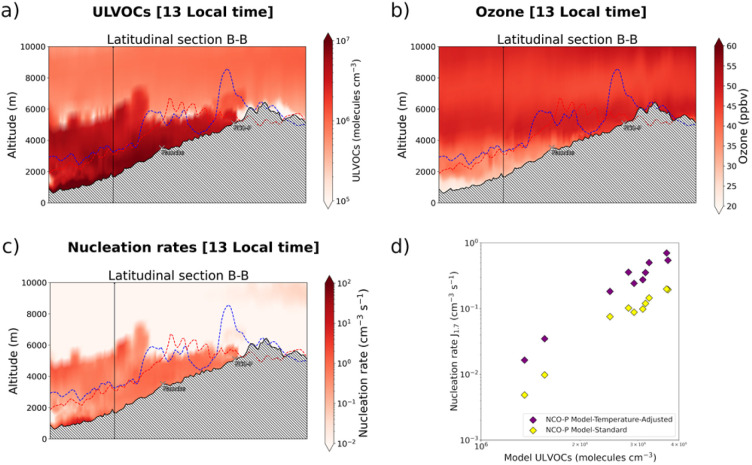
(a) Modelled ULVOCs concentrations (molecules per cm^3^ per s), (b) ozone concentrations along the latitudinal profile B–B (ppb), and (c) total nucleation rate (particles per cm^3^ per s), of the Khumbu valley at 13 local time. The effect of the temperature correction on the nucleation rate is presented as a scatterplot, (d).

Fig. 7 shows the modelled ULVOCs concentrations and nucleation rates, along with ozone concentrations and the temperature correction effect on the nucleation rates.

The data are reported at 13 local time for the 18th of December, the time when nucleation occurs in the modelled data. Modelled ULVOCs concentrations are in the 1 × 10^5^ molecules per cm^3^ and 1 × 10^7^ molecules per cm^3^ range. The highest concentrations can be observed at the entrance of the valley floor and within an altitude of about 5000 meters a.s.l., after which, the concentrations of ULVOCs abruptly decline to well below 1 × 10^6^ molecules per cm^3^. A closer look at these concentrations relative to the position of the western and eastern ridges of the Khumbu valley revealed another interesting pattern. Higher concentrations of ULVOCs can been seen above the valley ridges (red and blue lines in Fig. 7) for about 50 km, *i.e.* from the entrance of the valley at 27.3N to about around 27.75N, slightly south of the Namche station. These areas correspond to regions where most of the biogenic emissions are concentrated (Fig. 2). The elevated ULVOCs concentrations observed above the valley ridges in this section of the valley are likely due to the action of cross-valley winds injecting highly oxidized molecules to higher elevations (Fig. 2) where they nucleate depending on their concentration gradient. The nucleation was predicted to be entirely dominated by ion-induced pure biogenic nucleation, with the neutral channel close to negligible, and with the sulfuric acid and sulfuric acid–organics channels being negligible (not shown). Similarly, higher concentrations of ULVOCs along the valley floor from Namche on, are likely to be the result of the transport of those highly oxygenated molecules from the “biogenic reservoir” located in the valley entry (mostly below section A–A) all the way to NCO-P, where at 13 local time they nucleate.

**Fig. 8:**
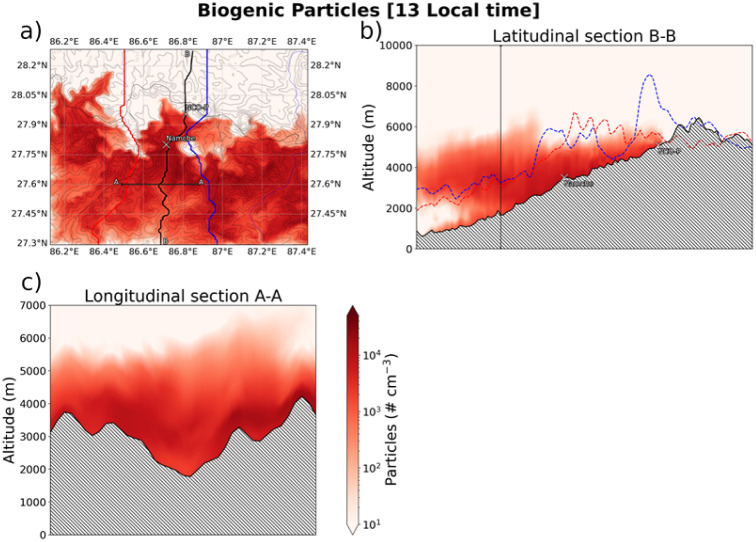
Geographical extent of biogenic particles produced in the Khumbu valley at 13 local time. Concentrations (particle per cm^3^) are reported along the longitudinal section A–A (panel c) and the latitudinal section B–B (panel b) of the Khumbu valley, and the over the whole inner high-resolution domain (panel a).

Finally, Fig. 8 shows the total number of biogenic particles as predicted by the model. Concentrations are reported at 13 local time, *i.e.* the arrival time of ULVOCs precursors at NCO-P and when the mountain circulation is at its maximum. Predicted concentrations are around 1 × 10^4^ particles per cm^3^, roughly distributed within a volume below 6000 m a.s.l. Additionally, the overall mechanism discussed so far seems to also affect the adjacent valleys on the left and right size of the Khumbu valley (top-left figure).

Under those assumptions, we cannot exclude that such a mechanism, with the “right” synoptic conditions present, can occur to the whole Himalayan chain.

## Limitations of the study

The intensity of the estimated biogenic emissions, the lack of an explicit autooxidation mechanism, and the proper representation of the mountain circulation, all add substantial uncertainties to the analysis.

Another major limitation in our study is the likely misrepresentation of any explicit anthropogenic influx from the Indo-Gangetic plain. The reasons for such intentional omissions were based on the extremely low anthropogenic influence observed at NCO-P during the campaign.^8^ However, if any anthropogenic material is present at the very entrance of the valley, it poses additional uncertainties. Therefore, the analysis presented here should be seen as representative of very pristine environmental conditions and further sensitive tests are planned to quantify the impact of anthropogenic emissions on the current results. In this regard, the first section of the valley that goes from the southern border of our domain, up to about the A–A section, could very likely be one of the best anthropogenic–biogenic interaction laboratories, and we encourage the idea of measurement campaigns located in the middle of the valley (in addition to the valley top). We refrained from artificially altering boundary conditions in absence of more detailed anthropogenic emissions data, at high-resolution, that we could supply to our coarser domain at 3 km. An increase, or decrease, of anthropogenic influxes, especially at the southern border of the domain, might dramatically change the overall picture presented here, both in terms of the availably of highly oxygenated molecules capable of nucleation, as well of the intensity of the valley-winds. Injection of inert black carbon, for example, could increase the condensational sink in the lower part of the valley floor, and alter the intensity of the valley-wind by changing the radiative balance over the valley.^33^ All those assumptions are planned to be tested in forthcoming analysis.

## Implications under future climate scenarios

**Fig. 9:**
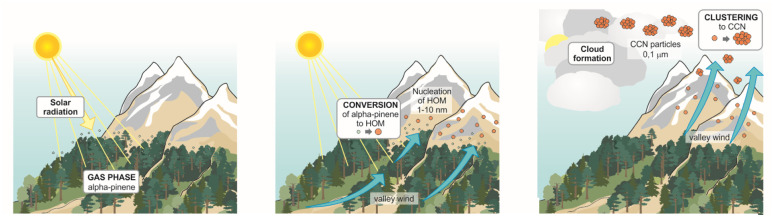
A schematic of the “Himalayan aerosol factory”. Courtesy of Sole Lätti (https://kuvittajat.fi/).

Fig. 9 recap the mechanism under discussion, *i.e.* the “Himalayan aerosol factory”.

If any climate relevance of the mechanism can be proven, and those particles can be detected in the free troposphere over the whole Himalayan chain, ideally augmentations and rearrangements of the vegetation in those regions coupled with increasing temperatures, could result in an increase of the biogenic aerosol loads in the free troposphere, and perhaps promote the formation of clouds. This perhaps can be thought as a natural geo-engineering process, which will increase the flux of natural aerosols from the ground, in contrast to a discontinuous elevated injection of anthropogenic seeds. Obviously, this Orwellian idea aims at provoking a discussion, to open a call for atmospheric scientists and civil and environmental engineers on such a topic, keeping in mind that “reducing the sources and increasing the sinks” must be our top priority for the years to come.

## Conclusions

A chemical transport modelling experiment was performed over the Himalayan chain, and specifically over the Khumbu valley and the Nepal Climate Observatory-Pyramid (NCO-P). The study aimed at supporting recent evidence of frequent pure biogenic nucleation events recorded at NCO-P and to explore its geographical extent. Results from this study align with the observational evidence confirming the key role of valley winds in transporting highly oxygenated molecules to the observational site, where they promote the formation of new particles. According to our model results, the presence of efficient nucleating gases and biogenic aerosol particles were predicted within an altitude of 5000 m a.s.l. The transport of the organic material was mediated by the valley-winds circulation, both by along-valleys and cross-ridges circulations, which develop during daytime as the thermal heating of the valley is initiated. The identical mechanism was also observed in the adjacent valleys included in the domain, suggesting that, under the proper synoptic conditions, it can possibly propagate to the whole Himalayan chain. Despite model limitations and uncertainties, above all the unknown interactions with the anthropogenic fluxes from the Indo-Gangetic plain, these results suggest that mountain regions can significantly impact the biogenic aerosol loads in the free troposphere. The climate implications for such mechanisms are still unknown, and further modelling studies are needed to properly quantify the significance of the “Himalayan aerosol factory”.

## Author contributions

GC led the work. AC and GC performed the model development. MB and BV performed the data analysis in collaboration with GC. VAS performed the initial WRF simulation. HG and IH supported the model development discussion. BB, AB, DZ, JM, PT, AM, JB, MK, DW, TN and FB supported the discussion.

## Conflicts of interest

There are no conflicts to declare.

## Data Availability

Data can be obtained from the corresponding authors.
